# A New Sythetic Hybrid (A_1_D_5_) between *Gossypium herbaceum* and *G*. *raimondii* and Its Morphological, Cytogenetic, Molecular Characterization

**DOI:** 10.1371/journal.pone.0169833

**Published:** 2017-02-10

**Authors:** Yuxiang Wu, Di Chen, Shuijin Zhu, Lufei Zhang, Lingjiao Li

**Affiliations:** 1 College of Agriculture, Shanxi Agricultural University, Taigu, Shanxi, China; 2 Department of Agronomy, College of Agriculture and Biotechnology, Zhejiang University, Hangzhou, Zhejiang, China; 3 Cotton Research Institute of Chinese Academy of Agricultural Science, Anyang, Henan, China; Wuhan University, CHINA

## Abstract

The diploid species *G*. *herbaceum* (A1) and *G*. *raimondii* (D5) are the progenitors of allotetraploid cotton, respectively. However, hybrids between *G*. *herbaceum* and *G*. *raimondii* haven’t been reported. In the present study, hybridization between *G*. *herbaceum* and *G*. *raimondii* was explored. Morphological, cytogenetic and molecular analyses were used to assess the hybridity. The interspecific hybrid plants were successfully obtained. Most of the morphological characteristics of the hybrids were intermediate between *G*. *herbaceum* and *G*. *raimondii*. However, the color of glands, anther cases, pollen and corolla, and the state of bracteoles in hybrids were associated with the *G*. *herbaceum*. The color of staminal columns and filaments in hybrids were associated with *G*. *raimondii*. Cytogenetic analysis confirmed abnormal meiotic behavior existed in hybrids. The hybrids couldn’t produce boll-set. Simple sequence repeat results found that besides the fragments inherited from the two parents, some novel bands were amplified in hybrids, indicating that potential mutations and chromosomal recombination occurred between parental genomes during hybridization. These results may provide some novel insights in speciation, genome interaction, and evolution of the tetraploid cotton species.

## Introduction

Cotton has important economic values in the world. It has been widely used in different fields, such as in the textile industry, in the production of oil and plant protein, and especially in taxonomic and evolutionary studies [1]. The cotton genus *Gossypium* includes 51 worldwide-distributed species, of which 46 are diploid (2n = 2x = 26) and the other five are tetraploid (2n = 4x = 52) [2]. Based on chromosomal pairing relationships and geographical distribution, the diploid species can be divided into eight subgenomes and designated as A to G and K [3]. Over the decades, various kinds of interspecific hybrids have been successfully established, such as *G*. *arboreum* × *G*. *raimondii* [4], *G*. *hirsutum* × *G*. *sturtianum* [5], *G*. *arboreum* × *G*. *bickii* [6], *G*. *hirsutum* × *G*. *klotzschianum* [7], *G*. *hirsutum* × *G*. *raimondii* (Benbouza et al, 2010), *G*. *hirsutum* × *G*. *trilobum* (D9) [8], *G*. *arboreum* × *G*. *anomalum* [9], *G*. *hirsutum* × *G*. *anomalum* [10], *G*. *herbaceum* × *G*. *australe* (G2) [11], etc. Currently, it is well accepted that hybridization plays a major role in cotton plant breeding due to the increase of the genetic diversity available and the production of new genotypes [12].

*Gossypium* allopolyploids, which are currently responsible for 95% of world cotton production and have high quality levels, are the result of the hybridization between two diploid species (A-genome and D-genome) approximately 1–2 million years ago [13, 14]. The diploid species *G*. *herbaceum* (A1) and *G*. *raimondii* (D5) are the progenitors of allotetraploid cotton [13]. They are highly similar to the relative tetraploid genomes at the molecular level and the EST-SSR sequences[15–17], and are important to study the evolution of cultivated cotton and fiber related genes. However, until now, hybrids between *G*. *herbaceum* and *G*. *raimondii* haven’t been reported.

In the present study, artificial interspecific hybrids (A_1_D_5_) were produced by crossing *G*. *herbaceum* (Genome A_1_A_1_) with *G*. *raimondii* (Genome D_5_D_5_). The main goal of this research was to investigate the breeding program of this new germplasm, to evaluate its morphology and cytology, as well as to identify its molecular characteristics. These results may provide novel insights in speciation, genome interaction, and chromosomal recombination occurred between parental genomes during hybridization.

## Materials and Methods

### Plant materials

The cultivated diploid cotton cultivar *G*. *herbaceum ‘*Redstar’ was used as the female parent to cross with a wild diploid cotton accession *G*. *raimondii* as the male parent. *G*. *herbaceum* cultivar was an inbreed line continuously self-pollinated for more than 20 generations. The pollen of *G*. *raimondii* was collected from the China National Wild Cotton Nursery in Sanya, China.

### Interspecific hybridization

Hybridization was conducted in the winter season of 2012–2013 in Sanya, China. The flowers for the female parent, which were going to bloom in the next day, were emasculated and stigmas were covered with waxed tubes to avoid cross-pollination. During the blooming period (8:00 AM to 12:00 PM) of the next morning, the emasculated female flowers were pollinated three times with *G*. *raimondii* pollen. The crossed seeds were harvested from the female plants and planted in nutrition pots. Two week old-seedlings were transplanted into the field at Sanya on May 15, 2014.

### Morphological analyses

Fully expanded leaves from the same position on the parents and the hybrid plants were characterized, including the shape, size, presence of pubescence and gland color. During flower development, the expanded flower morphological traits of the putative hybrid plants were compared with those of their parents, including bracteoles, calyx, corolla, stigma, androecium, pollen and capsule.

### Cytogenetic observation

Anthers at different development stages were collected from immature flower buds of the hybrid plants and fixed in freshly prepared Carnoy's fluid (ethanol: acetic acid = 3:1 v/v) [11] immediately. After 24 hours, the fixed anthers were transferred to 70% ethanol and stored at 4°C for cytogenetic observation. The fixed anthers were then stained on slides using Carbol fuchsin solution for microscopic evaluation. Photographs were taken from freshly prepared slides using an Olympus BX60 microscope with automatic camera. Meiosis was studied using a minimum of 500 pollen mother cells (PMCs).

### Molecular identification

Genomic DNA from three hybrid plants and their parental accessions were extracted as previously described with minor modifications [18]. Briefly, DNA was extracted from young leaves using CTAB method and quantified using agarose gel electrophoresis. DNA amplification was carried out in a total volume of 20 μL containing 2 μL 10 × buffer, 1.6 μL MgCl_2_ (25 mM), 0.2 μL dNTPs (10 mM), 6 μL template DNA (50 ng /μL), 2 μL forward and reverse primers (2.5 μM), 0.2 μL Taq polymerase (5 U/μL, Sangon), and 8 μL ddH_2_O. Nine pairs of simple sequence repeat (SSR) primers were used for amplification (Table 1). PCR amplification was performed in a DNA Mastercycler (Eppendorf, Germany), using an initial denaturing at 94°C for 4 minutes; followed by 35 cycles of pre-denaturation at 94°C for 3 minutes; denaturation at 94°C for 50 seconds; annealing at 58°C for 50 seconds; elongation at 72°C for 2 minutes; and a final extension at 72°C for 10 minutes. Amplified fragments (20 μL) were separated by PAGE and resolved by silver staining.

**Table 1:** Sequence information of SSR polymorphism primers (from http://www.cottonmarker.org/cgi-bin/cmd_search_marker.cgi).

Primer number	Forword sequence (5’-3’)	Reverse sequence (5’-3’)
BNL4108	TCCACCATTCCCGTAAATGT	TGGCCAAGTCATTAGGCTTT
BNL4053	TGAAGGCTTTGAAGCAAACA	AAGCAAGCACCAAGTTAGCC
NAU2026	GAATCTCGAAAACCCCATCT	ATTTGGAAGCGAAGTACCAG
NAU1355	ATCTGTTTACGCCACTCTCC	CCAGCCTTTGACATTTTTCT
NAU1169	GGGTAGTAGCTTTTATGATAGGG	CCATTCCTTCCCCTAATTCT
NAU1157	GAGTTTGGTTCTGGGTTGAG	GATCCTTTTCATCTCCTCCA
NAU1052	CGCAGATAAAGGATGGATTT	AGAGCTGGAGGACATAACAAA
NAU1042	CATGCAAATCCATGCTAGAG	GGTTTCTTTGGTGGTGAAAC
NAU1164	CCAACGCTAATTCTACCTCCT	GCGGGTAATTGTAGTACATGC

## Results

### Morphological characteristics of AD interspecific hybrid

**Fig 1:**
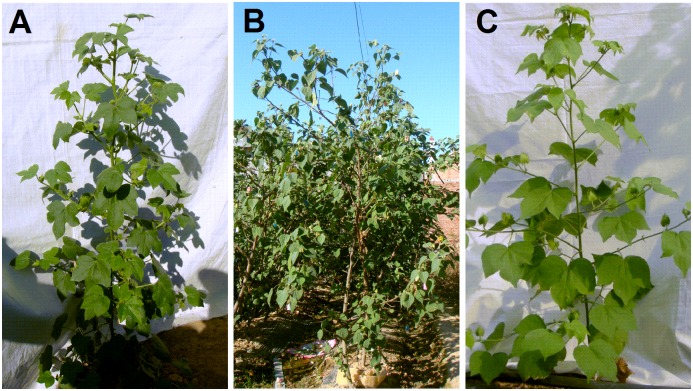
The images of *Gossypium herbaceum* (A), *G*. *raimondii* (B), *Gossypium herbaceum* × *G*. *raimondii* F1 hybrid (C).

**Fig 2:**
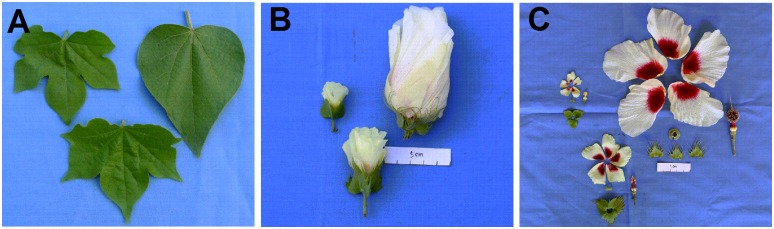
Morphological characteristics of *G*. *herbaceum*(upper, left), *G*. *raimondii* (upper, right) and *Gossypium herbaceum* × *G*. *raimondii* F1 hybrid (lower). A) Leaf. B) Flower. C) Petals, bracts, calyxs, staminal column and filaments.

**Fig 3:**
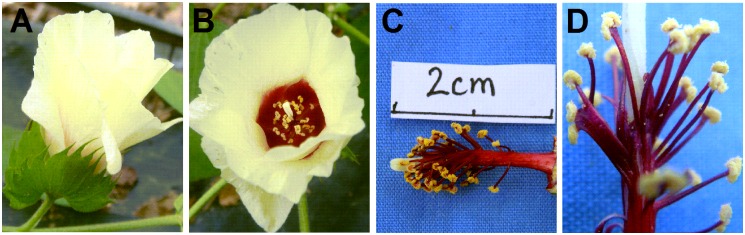
Flower characteristics of *Gossypiumherbaceum* × *G*. *raimondii* F1 hybrid. A-B) Flower. C-D) Staminal column and filaments.

To understand the morphological differences between AD interspecific hybrid and parents, more than 500 crossed flowers were investigated. Among them, 11 hybrid bolls with 1–3 seeds per capsule were harvested. Among 27 hybrid seeds obtained, 24 were found to be empty and failed to germinate. Three well-developed hybrid seeds germinated and eventually developed into vigorous hybrid plants. The hybrid plants exhibited prolific growth with extensive monopodial branching, which was similar to *G*. *herbaceum* and *G*. *raimondii* (Fig 1). The plant height of *G*. *herbaceum* varied from 50 cm to 70 cm and *G*. *raimondii* could reach a height of 5.0 m. Meanwhile, the main stem diameters of *G*. *herbaceum* and *G*. *raimondii* were 1.5 cm and 5.0 cm respectively. The average height of hybrids was 3.0 m and the average diameter of their main stems was 3.0 cm. Both parameters were in-between two parents. Flower buds of the hybrids appeared at 45 d after planting and they produced flowers profusely and sequentially. *G*. *herbaceum* can flower in a whole year in Sanya, China. However, the flowering time of hybrids was from November in the first year to May in the second year, which was similar to *G*. *raimondii*. In addition, many morphological characteristics of the hybrids were also intermediate between *G*. *herbaceum* and *G*. *raimondii*, such as the size and shape of leaves (Fig 2A), flowers (Fig 2B), petals, bracts, calyxes, the number of acuminate lobes and the length of androecium (Fig 2C). The color of gland, anther, pollen and corolla in hybrids was yellow, which was similar to that in *G*. *herbaceum* (Figs 2C and 3). Bracteoles united at base in hybrids were also associated with *G*. *herbaceum* (Fig 2C). However, the color of staminal column and filaments in hybrids was deep red, which was associated with *G*. *raimondii* (Figs 2C, 3C and 3D). Meanwhile, anthers and the number of filaments in hybrids were distinct from those in the parents whose filaments were wide and flatted with clustering of anthers (Figs 2C, 3C and 3D). Hybrids showed no boll-set. The main morphological characters of *G*. *herbaceum*, *G*. *raimondii*, and the F_1_ hybrids are summarized in Table 2.

**Table 2:** Comparison of main characters of *G*. *herbaceum*, *G*. *raimondii*, and *G*.*herbaceum×G*. *raimondii*.

Organs	*G*. *herbaceum*	*G*. *herbaceum*×*G*. *raimondii*	*G*. *raimondii*
Leaf	22–95 mm length, 25–100 mm width; 5 lobed	20–110 mm length, 15–125 mm width; entire or 2–5 lobed	12–170 mm length, 12–150 mm width; entire
Gland color	Yellow	Yellow	Cream
Bracteoles	15 mm length, 14 mm width; 7–8 ebetate lobes, 1–6 mm length, united at base	26 mm length, 25 mm width; 10–12 acuminate lobes, 2–11 mm length, united at base	40 mm length, 35 mm width; 15–16 acuminate lobes, 5–20 mm length, free at base
Calyx	Tube, 2–3 mm length; 7–8 lobes, 0.5–1.0 mm length	Tube, 3–5 mm length; 5 lobes, 1–2 mm length	Tube, 5–6 mm length; united
Corolla	Yellow; small petal spot, deep red	Yellow; large petal spot, deep red	Cream; very large petal spot, deep red
Stigma	Light yellow; 3–5 mm length	Light yellow; 4–7 mm length	Light yellow; 3–5 cm length
Androecium	Light yellow staminal column and filaments; yellow anther cases	Deep red staminal column and filaments; yellow anther cases	Deep red staminal column and filaments; red anther cases
Pollen	Yellow	Yellow	Cream
Capsule	3 locules; 1–6 seeds per locule	No boll setting	3 locules; 2–3 seeds per locule
Pubescence	Puberulent	Canescent	Canescent

### Cytogenetic observation

**Fig 4:**
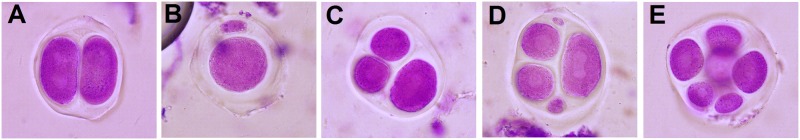
The normal and abnormal meiosis behavior of *Gossypium herbaceum* × *G*. *raimondii* F1 hybrid. A) Normal dyad. B) Unequal separation of dyad. C) Non-synchronized division of dyad in meiosis II. D) Unequal division with various abnormal micronuclei. E) Abnormal polyads.

**Fig 5:**
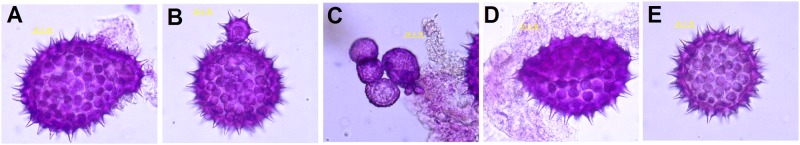
Various abnormal pollen grains after meiosis of *Gossypium herbaceum* × *G*. *raimondii* F1 hybrid. A) Big oval-shaped pollen. B) Small under-developed pollen. C) Deformed pollen. D) Cracked pollen. E) Normal pollen.

To further investigate the hybrid specialty at the cytogenetic level, meiosis in hybrids was observed. Some meiotic stages of the hybrids exhibited normal behavior, including normal dyad of PMC (Fig 4A), normal tetrad of PMC, and normal pollen grains (Fig 5E). But abnormal meiosis behavior in PMC from hybrids also existed, such as unequal separation of dyad (Fig 4B), the occurrence of triad partly from non-synchronized separation of dyad (Fig 4C), unequal division with abnormal unbalanced micronucleus formation (Fig 4D), and various abnormal polyads (Fig 4E) in telophase II. These results are due to non-synchronous chromosome separation at anaphase I in hybrid meiosis. Of 500 multispores, there were 104 dyads (20.80%), 44 triads (8.80%), 197 tetrads (39.40%) and 155 other polyads (31.00%) respectively (Table 3). Among the 197 tetrads, 120 were normal tetrads (60.91%) and 77 were abnormal tetrads (39.09%). Abnormal tetrads could not form into normal pollen grains, so various abnormal pollen grains were observed, including big oval-shaped pollen, small under-developed pollen, deformed pollen, cracked pollen and so on (Fig 5). Among the 500 selected pollen grains, only 292 pollen grains (58.40%) appeared to be normal.

**Table 3:** Number of multispores in telophase II of AD hybrid meiosis.

Shape	Dyad	Triad	Tetrad	Polyad	Total
No	104	44	197	155	500
%	20.80	8.80	39.40	31.00	100

### Molecular analysis

**Fig 6:**
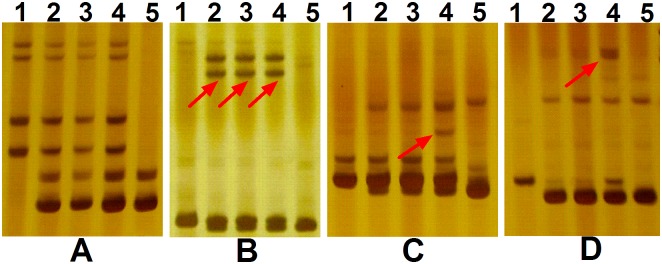
Amplification results for three *Gossypium herbaceum* × *G*. *raimondii* F1 hybrids and two parents using 4 representative SSR primer pairs. A) BNL4053. B) NAU2026. C) NAU1157. D) NAU1164 respectively.1-5: *G*. *herbaceum*, hybrid plant 1, hybrid plant 2, hybrid plant 3, and *G*. *raimondii*, respectively. The novel bands produced in hybrid plants were indicated by arrows.

To better understand the differences between the hybrid and parents on a molecular level, SSR analysis of the three hybrids and the two parents was performed using nine SSR primer pairs (Table 1) and all yielded microsatellite products. Among these products, the bands amplified by primer BNL4053 in the three hybrids exhibited the complementary contribution from the two parents (Fig 6A). Other than the bands inherited from the two parents, novel bands were produced in hybrid plants using primers NAU2026, NAU1157 and NAU1042. Novel bands were amplified in all three hybrids by primer NAU2026 (Fig 6B, arrow showing). Furthermore, two novel bands in hybrid 3 were produced by primers NAU1157 (Fig 6C, arrow showing) and NAU1164 (Fig 6D, arrow showing) respectively, indicating that there were mutations and chromosomal recombination occurred between parental genomes during hybridization.

## Discussion

Polyploidy is a prominent process in plant evolution, especially among angiosperm species [19–22]. All angiosperms are believed to have experienced at least one round of whole-genome duplication during their evolution [23]. The reunion of genomes through hybridization and allopolyploidy may account for 2–4% of speciation events in flowering plants and 7% in ferns [24]. Many of the world’s leading crops are polyploids, including genus *Gossypium* [25]. The genus *Gossypium* has been widely used for investigating emergent consequences of polyploidy. *Gossypium* allopolyploids are the result of hybridization between two diploid species (A-genome and D-genome), which occurred approximately 1–2 million years ago [13, 14]. Due to geographical isolation and genetic differentiation, the different tetraploid species are evolved [26, 27]. Compared with the cotton produced by parallel improvement of A genome diploid cottons, domestication and breeding of AD tetraploid cottons have higher fiber yield and quality levels. This comparison implied that the merger of two genomes with different evolutionary histories in a common nucleus can offer unique avenues for phenotypic response to selection [28]. It has been demonstrated that the diploid species *G*. *herbaceum* (A1) and *G*. *raimondii* (D5) are the progenitors of allotetraploid cotton [14]. In the present study, the new interspecific hybrids (A_1_D_5_) between *G*. *herbaceum* and *G*. *raimondii* was first produced (Fig 1), which provided the novel insights of genome interaction and speciation as well as its potential applications.

Phenotypic variations have been found in the genome-wide changes resulting from hybridization. The most common phenotypes of AD hybrids were intermediates between the two parents with some other variations[29–31]. The changes in the morphology characteristics of the new AD interspecific hybrid in the present study are consistent with the above reports. Most of the morphological characteristics of the hybrids were found to be intermediate between *G*. *herbaceum* and *G*. *raimondii*. However, the color of glands, anther cases, pollen and corolla, and the state of bracteoles in hybrids were associated with the *G*. *herbaceum*. The color of the staminal columns and filaments in hybrids was associated with *G*. *raimondii* (Figs 2 and 3, Table 2). It has been reported that the newly formed hybrids may be inviable or sterile [32]. In this study, the AD hybrid between *G*. *herbaceum* and *G*. *raimondii* was also sterile, which supported the above conclusion. Meiosis observation showed that various abnormal meiotic behaviors in PMC and abnormal pollen grains existed in the hybrids (Figs 4 and 5, Table 3), which may be involved in the sterilization of the hybrids.

The phenotypic variation of the hybrids may be associated with a variety of factors, including novel gene combinations, chromosomal rearrangements, etc.[30, 33–39]. DNA molecular markers have been widely used in genetic analyses, breeding and investigations of genetic diversity [26]. Since it has been discovered that microsatellites markers are evenly distributed along chromosomes in cotton, SSRs have been widely used to monitor the introgression of alien chromosome fragments in interspecific hybrids [1, 40]. The transcriptional phenotypes of first generation hybrids should predominantly reflect the basic interaction of parental genomes and their endogenous regulatory factors [41]. Moreover, when A and D genomes combined into a single nucleus, they shared gene regulatory factors, which led to novel regulatory processes and changes in regulatory networks. These novel interactions were considered as a genome shock that accompanies polyploidization and might provide a new source of genetic variation [35, 42, 43]. Furthermore, the novel mutational bands have been amplified in hybridization of *Brassica oleracea var*. *alboglabra* and *B*. *rapa var*. *purpurea*, suggesting interspecific hybridization may play an important role in the fast evolution of vegetable crops [44]. Our present study also observed the novel bands in the hybrids (Fig 6B, 6C and 6D) from SSR analyses, implying that potential chromosomal combination and mutations also occurred during AD interspecific hybridization. Our findings strongly supported the previous hypothesis that interspecific hybridization is an important driving force to develop new mutants and novel phenotypes during plant evolution [45–47]. Furthermore, considering the difficulty of interspecific hybridization to get AD hybrids in this study, we inferred that there might be high-level genome-wide mutations and/or incompatibility between interacting chromosomes during hybridization to cause the high rate of reproductive failure of hybrids.

In conclusion, artificial interspecific hybrids (A_1_D_5_) were first produced in the present study by crossing *G*. *herbaceum* with *G*. *raimondii*. The breeding program of this new germplasm, its morphological and cytological characteristics, and their molecular identification were analyzed. The novel bands amplified in hybrids indicated the chromosomal combination and mutations occurring in AD interspecific hybridization. These results shed new light on speciation, genome interaction, and evolution of the tetraploid cotton species.

## Supporting Information

S1 Fig Immatured boll. Setting boll on the mutant branch after chromosome doubling by colchicine treatment of this new sythetic hybrid (A_1_D_5_).
(TIF)

S2 Fig Matured boll. Matured empty boll without developed seeds on the mutant branch after chromosome doubling by colchicine treatment of this new sythetic hybrid (A_1_D_5_).
(TIF)

## References

[1] Benbouza H, Lacape J, Jacquemin J, Courtois B, Diouf F, Sarr D, et al Introgression of the low-gossypol seed & high-gossypol plant trait in upland cotton: Analysis of [(Gossypium hirsutum× G. raimondii) ²× G. sturtianum] trispecific hybrid and selected derivatives using mapped SSRs. Molecular breeding. 2010;25(2):273–86.

[2] Fryxell P. A revised taxonomic interpretation of Gossypium L.(Malvaceae). Rheedea. 1992;2(2):108–65.

[3] Stewart J. Potential for crop improvement with exotic germplasm and genetic engineering. In: Constable GA, Forrester NW, editors. Challenging the Future: Proceedings of the World Cotton Research Conference-1 CSIRO, Melbourne, Australia; 1994. Pp. 313–327.

[4] Endrizzi J, Phillips L. A hybrid between Gossypium arboreum L. and G. raimondii Ulb. Canadian Journal of Genetics and Cytology. 1960;2(4):311–9.

[5] Muramoto H. Hexaploid cotton: some plant and fiber properties. Crop Science. 1969;9(1):27–9.

[6] Li B, Zhang B, Zhang X, Niu Y. Studies on the Hybrid between G. arboreum L and G. bickii Prokh. Acta Genetica Sinica. 1987;14(2):121–6.

[7] Sun Y, Zhang X, Nie Y, Guo X, Jin S, Liang S. Production and characterization of somatic hybrids between upland cotton (Gossypium hirsutum) and wild cotton (G. klotzschianum Anderss) via electrofusion. Theoretical and applied genetics. 2004;109(3):472–9. 10.1007/s00122-004-1663-3 15114473

[8] Yu X, Chu B, Liu R, Sun J, Brian JJ, Wang H, et al Characteristics of fertile somatic hybrids of G. hirsutum L. and G. trilobum generated via protoplast fusion. Theoretical and applied genetics. 2012;125(7):1503–16. 10.1007/s00122-012-1929-0 22777361

[9] Newaskar GS, Chimote VP, Mehetre SS, Jadhav AS. Interspecific hybridization in Gossypium L.: characterization of progenies with different ploidy-confirmed multigenomic backgrounds. Plant Breeding. 2013;132(2):211–6.

[10] Zhang X, Zhai C, He L, Guo Q, Zhang X, Xu P, et al Morphological, cytological and molecular analyses of a synthetic hexaploid derived from an interspecific hybrid between Gossypium hirsutum and Gossypium anomalum. The Crop Journal. 2014;2(5):272–7.

[11] Liu Q, Chen Y, Wang Y, Chen J, Zhang T, Zhou B. A New Synthetic Allotetraploid (A1A1G2G2) between Gossypium herbaceum and G. australe: Bridging for Simultaneously Transferring Favorable Genes from These Two Diploid Species into Upland Cotton. Plos One. 2015;10(4):e0123209 10.1371/journal.pone.0123209 25879660 PMC4400159

[12] Noormohammadia Z, Taghavia E, Foroutanb M, Sheidaib M, Alishahc O. Structure analysis of genetic diversity in tetraploid and diploid cotton genotypes. Inter. J. Plant Animal Environ. Sci. 2013;3:79–86

[13] Wendel JF, Cronn RC. Polyploidy and the evolutionary history of cotton. Advances in agronomy. 2003;78:139–86.

[14] Brubaker C, Paterson A, Wendel J. Comparative genetic mapping of allotetraploid cotton and its diploid progenitors. Genome. 1999;42(2):184–203.

[15] Grover CE, Kim H, Wing RA, Paterson AH, Wendel JF. Incongruent patterns of local and global genome size evolution in cotton. Genome Res. 2004;14(8):1474–82. 10.1101/gr.2673204 15256507 PMC509256

[16] Senchina DS, Alvarez I, Cronn RC, Liu B, Rong J, Noyes RD, et al Rate variation among nuclear genes and the age of polyploidy in Gossypium. Molecular Biology & Evolution. 2003;20(4):633–43. 12679546 10.1093/molbev/msg065

[17] Zhu HY, Zhang TZ, Yang LM, Guo WZ. EST-SSR sequences revealed the relationship of D-genome in diploid and tetraploid species in Gossypium. Plant Science. 2009;176(3):397–405.

[18] Paterson AH, Brubaker CL, Wendel JF. A rapid method for extraction of cotton (Gossypium spp.) genomic DNA suitable for RFLP or PCR analysis. Plant Molecular Biology Reporter. 1993;11(2):122–7.

[19] Comai L. The advantages and disadvantages of being polyploid. Nature Reviews Genetics. 2005;6(11):836–46. 10.1038/nrg1711 16304599

[20] Flagel LE, Wendel JF. Evolutionary rate variation, genomic dominance and duplicate gene expression evolution during allotetraploid cotton speciation. New Phytol. 2010;186(1):184–93. 10.1111/j.1469-8137.2009.03107.x 20002320

[21] Leitch AR, Leitch IJ. Genomic plasticity and the diversity of polyploid plants. Science. 2008;320(5875):481–3. 10.1126/science.1153585 18436776

[22] Wendel JF. Genome evolution in polyploids. Plant Mol Biol. 2000; 42(1): 225–249. 10688139

[23] Xiong Z, Gaeta RT, Pires JC. Homoeologous shuffling and chromosome compensation maintain genome balance in resynthesized allopolyploid Brassica napus. Proc Natl Acad Sci U S A. 2011;108(19):7908–13. 10.1073/pnas.1014138108 21512129 PMC3093481

[24] Otto SP, Whitton J. Polyploid incidence and evolution. Annual review of genetics. 2000;34(1):401–37. 10.1146/annurev.genet.34.1.401 11092833

[25] Hilu K. Polyploidy and the evolution of domesticated plants. American Journal of Botany. 1993:1494–9.

[26] Wu Y-X, Daud M, Chen L, Zhu S-J. Phylogenetic diversity and relationship among Gossypium germplasm using SSRs markers. Plant systematics and evolution. 2007;268(1–4):199–208.

[27] Yu-xiang W, Jin-hong C, Qiu-ling H, Shui-jin Z. Parental origin and genomic evolution of tetraploid Gossypium species by molecular marker and GISH analyses. Caryologia. 2013;66(4):368–74.

[28] Jiang C, Wright RJ, El-Zik KM, Paterson AH. Polyploid formation created unique avenues for response to selection in Gossypium (cotton). Proc Natl Acad Sci U S A. 1998;95(8):4419–24. 9539752 10.1073/pnas.95.8.4419 PMC22504

[29] Gaeta RT, Pires JC, Iniguezluy F, Leon E, Osborn TC. Genomic Changes in Resynthesized Brassica napus and Their Effect on Gene Expression and Phenotype. Plant Cell. 2007;19(11):3403–17. 10.1105/tpc.107.054346 18024568 PMC2174891

[30] Ni Z, Kim ED, Ha M, Lackey E, Liu J, Zhang Y, et al Altered circadian rhythms regulate growth vigour in hybrids and allopolyploids. Nature. 2009;457(7227):327–31. 10.1038/nature07523 19029881 PMC2679702

[31] Pires JC, Lim KY, Kovarik A, Matyasek R, Boyd A, Leitch AR, et al Molecular cytogenetic analysis of recently evolved Tragopogon (Asteraceae) allopolyploids reveal a karyotype that is additive of the diploid progenitors. American Journal of Botany. 2004;91(7):1022 10.3732/ajb.91.7.1022 21653458

[32] STEBBINS GL. The inviability, weakness, and sterility of interspecific hybrids. Advances in genetics. 1957;9:147–215. 10.1016/s0065-2660(08)60162-5 13520442

[33] Beaulieu J, Jean M, Belzile F. The allotetraploid Arabidopsis thaliana-Arabidopsis lyrata subsp. petraea as an alternative model system for the study of polyploidy in plants. Molecular Genetics and Genomics. 2009;281(4):421–35. 10.1007/s00438-008-0421-7 19148683

[34] Kashkush K, Feldman M, Levy AA. Gene loss, silencing and activation in a newly synthesized wheat allotetraploid. Genetics. 2002;160(4):1651–9. 11973318 10.1093/genetics/160.4.1651 PMC1462064

[35] Rieseberg LH. Evolution: replacing genes and traits through hybridization. Curr Biol. 2009;19(3): 119–22. 10.1016/j.cub.2008.12.016 19211049

[36] Rowe HC, Rieseberg LH. Genome-scale transcriptional analyses of first-generation interspecific sunflower hybrids reveals broad regulatory compatibility. BMC Genomics. 2013;14:342 10.1186/1471-2164-14-342 23701699 PMC3679827

[37] Shaked H, Kashkush K, Ozkan H, Feldman M, Levy AA. Sequence elimination and cytosine methylation are rapid and reproducible responses of the genome to wide hybridization and allopolyploidy in wheat. Plant Cell. 2001;13(8):1749–59. 11487690 10.1105/TPC.010083 PMC139131

[38] Shan X, Liu Z, Dong Z, Wang Y, Chen Y, Lin X, et al Mobilization of the active MITE transposons mPing and Pong in rice by introgression from wild rice (Zizania latifolia Griseb.). Mol Biol Evol. 2005;22(4):976–90. 10.1093/molbev/msi082 15647520

[39] Swanson-Wagner RA, DeCook R, Jia Y, Bancroft T, Ji T, Zhao X, et al Paternal dominance of trans-eQTL influences gene expression patterns in maize hybrids. Science. 2009;326(5956):1118–20. 10.1126/science.1178294 19965432

[40] Siu L, Saha S, Stelly D, Burr B, Cantrell R. Chromosomal assignment of microsatellite loci in cotton. Journal of Heredity. 2000;91(4):326–32. 10912681 10.1093/jhered/91.4.326

[41] Grover CE, Gallagher JP, Szadkowski EP, Yoo MJ, Flagel LE, Wendel JF. Homoeolog expression bias and expression level dominance in allopolyploids. New Phytol. 2012;196(4):966–71. 10.1111/j.1469-8137.2012.04365.x 23033870

[42] Madlung A, Wendel JF. Genetic and epigenetic aspects of polyploid evolution in plants. Cytogenet Genome Res. 2013;140(2–4):270–85. 10.1159/000351430 23751292

[43] Osborn TC, Pires JC, Birchler JA, Auger DL, Chen ZJ, Lee HS, et al Understanding mechanisms of novel gene expression in polyploids. Trends Genet. 2003;19(3):141–7. 12615008 10.1016/s0168-9525(03)00015-5

[44] Zhang X, Liu T, Li X, Duan M, Wang J, Qiu Y, et al Interspecific hybridization, polyploidization, and backcross of Brassica oleracea var. alboglabra with B. rapa var. purpurea morphologically recapitulate the evolution of Brassica vegetables. Sci Rep. 2016;6:18618 10.1038/srep18618 26727246 PMC4698638

[45] Barton NH. The role of hybridization in evolution. Mol Ecol. 2001;10(3):551–68. 11298968 10.1046/j.1365-294x.2001.01216.x

[46] Jiao Y, Wickett NJ, Ayyampalayam S, Chanderbali AS, Landherr L, Ralph PE, et al Ancestral polyploidy in seed plants and angiosperms. Nature. 2011;473(7345):97–100. 10.1038/nature09916 21478875

[47] Soltis PS, Soltis DE. The role of genetic and genomic attributes in the success of polyploids. Proc Natl Acad Sci U S A. 2000;97(13):7051–7. 10860970 10.1073/pnas.97.13.7051 PMC34383

