# Supplementary figures and images for: A New Sythetic Hybrid (A1D5) between Gossypium herbaceum and G. raimondii and Its Morphological, Cytogenetic, Molecular Characterization
Source: PLoS One. 2017 Feb 10;12(2):e0169833. doi: 10.1371/journal.pone.0169833 (PMC5302248; doi:10.1371/journal.pone.0169833)

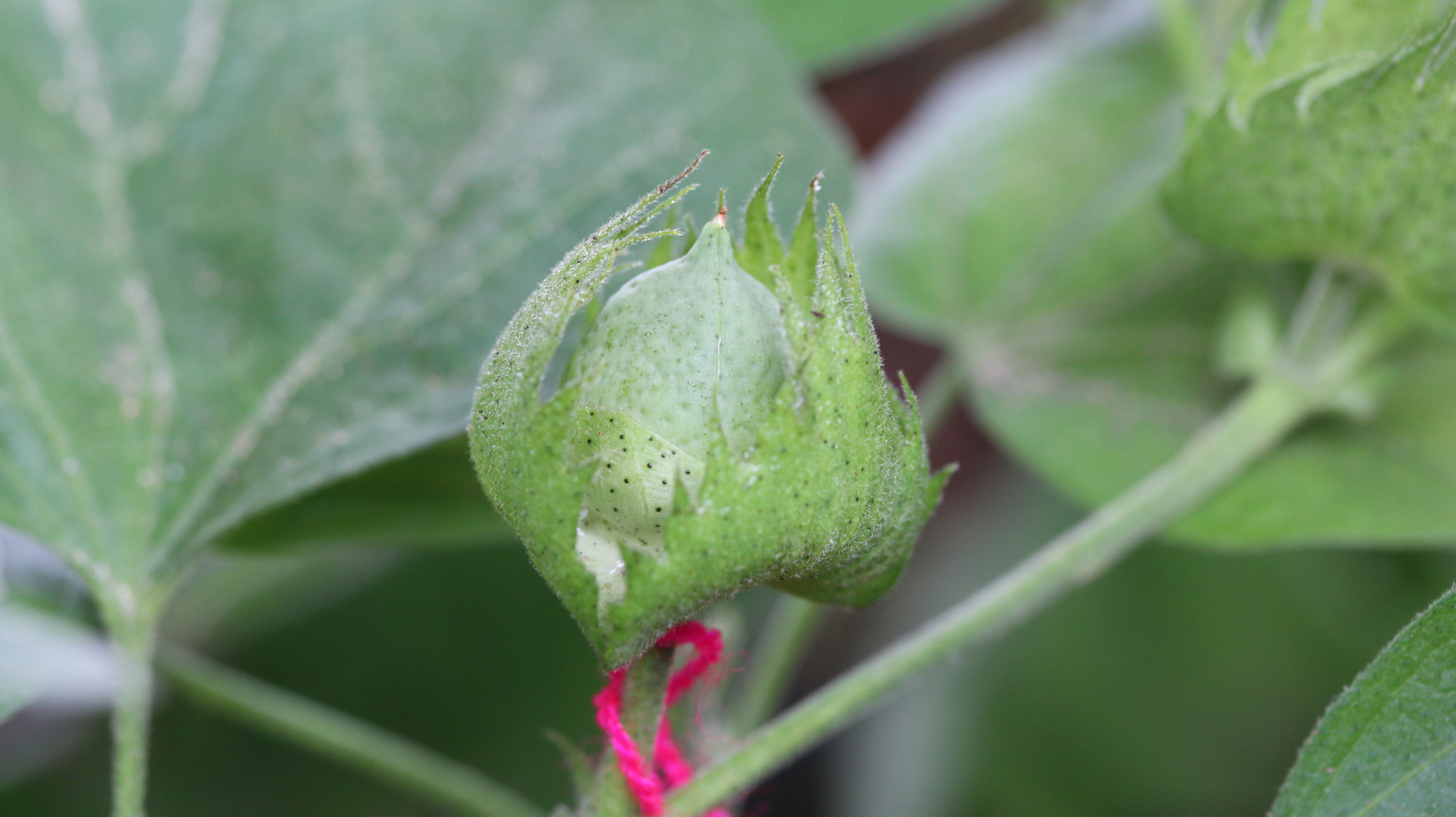

Supplement: S1 Fig — Setting boll on the mutant branch after chromosome doubling by colchicine treatment of this new sythetic hybrid (A1D5). (TIF) [file pone.0169833.s001.tif]

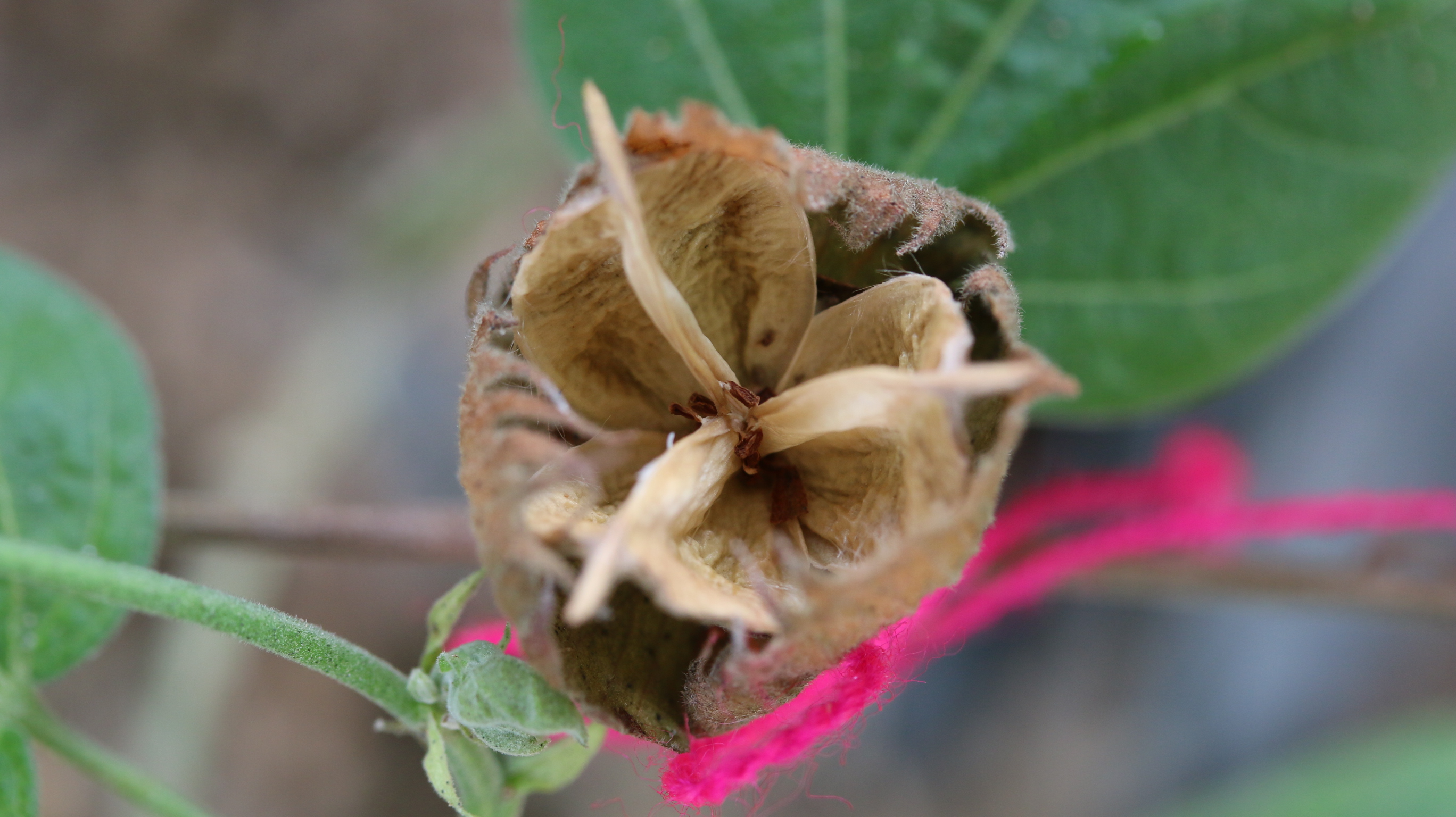

Supplement: S2 Fig — Matured empty boll without developed seeds on the mutant branch after chromosome doubling by colchicine treatment of this new sythetic hybrid (A1D5). (TIF) [file pone.0169833.s002.tif]
